# An Analysis of Societal Determinant of Anemia among Adolescent Girls in Azad Jammu and Kashmir, Pakistan

**DOI:** 10.1155/2020/1628357

**Published:** 2020-01-21

**Authors:** Nazneen Habib, Saif-Ur-Rehman Saif Abbasi, Wajid Aziz

**Affiliations:** ^1^Department of Sociology, International Islamic University, Islamabad, Pakistan; ^2^Department of Sociology, The University of Azad Jammu & Kashmir, Muzaffarabad 13100, Pakistan; ^3^Department of CS&IT, The University of Azad Jammu & Kashmir, Muzaffarabad 13100, Pakistan; ^4^Department of Computer Science & Artificial Intelligence, University of Jeddah, Jeddah, Saudi Arabia

## Abstract

Societal determinants of health are of recognized importance for understanding the causal association of society and health of an individual. Iron deficiency anemia (IDA) is a challenging public health problem across the globe instigating from a broader sociocultural background. It is more prevalent among pregnant women, children under the age of five years, and adolescent girls. Adolescent girls are vulnerable to develop IDA because of additional nutritional demand of the body needed for growth spurt, blood loss due to onset of menarche, malnourishment, and poor dietary iron intake. In this study, we explore the societal determinants of anemia among adolescent girls in Azad Jammu and Kashmir (AJK), Pakistan. A cross-sectional study was conducted in the Muzaffarabad division of AJK on randomly selected 626 adolescent girls. The data were collected using a pretested self-administered interview schedule comprising mainly closed-ended questions with a few open-ended questions. Descriptive statistics was computed for describing the data, and bivariate regression and logistic regression were used to determine the association of anemia with its societal determinants. Multiple linear regression is used to determine the relationship of different determinants (independent variables) with the hemoglobin level (dependent variable) of the respondents. The prevalence of anemia among adolescent girls is 47.9%, of which 47.7% have mild anemia, 51.7% have moderate anemia, and 5.7% have severe anemia, which reveals that anemia is a severe public health problem among adolescent girls in the study area. The findings aver that anemia occurrence was significantly associated with the respondent's and her parental education, economic well-being, prevalence of communicable diseases, menstrual disorder, exercise habits, meals regularity, and type of sewerage system.

## 1. Introduction

Anemia is a widespread public health problem across the globe that accounts for majority of nutritional problems and is principally caused by iron deficiency [1]. Its prevalence is substantially higher among the people residing in the developing countries (89%) because of low socioeconomic status and impoverished access to health care [2–4]. Anemia affects around 2 billion of the world's population, with consequences not only on the human health but also on the social and economic development [3, 4]. In South Asian countries, anemia is a problem of moderate (20.0–39.9%) or a severe (≥40%) significance from public health perspective among preschool children as well as in pregnant and nonpregnant women [5, 6].

Anemia falls into sever public health category in Pakistan, which is equally prevalent among pregnant women (50%, 41 to 58) and nonpregnant women (51%, 43 to 59) [7, 8]. In Azad Jammu and Kashmir (AJK), anemia prevalence is 41.0%, whereas 25% nonpregnant and 32% pregnant women are iron deficient [7]. Recent studies reported that anemia is the most prevalent micronutrient deficiency among adolescent girls in Pakistan [9–11]. The nutritional interventions taken during the adolescent period before females get married and enter motherhood can be helpful to avoid anemia among women of reproductive age (WRA).

Adolescence is the period of aging between 10 and 19 years [12], during which body needs for both macronutrient and micronutrient are considerably high because of peak pubertal development, growth spurt, and physical activity. The daily iron need increases twofolds to threefolds during adolescence for both boys and girls, and after the menarche, iron need continues to remain high among adolescent girls because of blood loss during menstruation [13], which increases the risk of nutritional anemia among them. Nutritional anemia has negative irreversible consequences on the growth, cognitive development, working capability, immune system that can lead to infections, and reproductive years of life [14, 15].

Numerous studies conducted across the globe highlighted that anemia is a public health problem among adolescent girls [9–11, 14–16]. In Pakistan, studies conducted on anemia among the adolescent girls [9–11] are not only few but also did not address societal determinants and the impact of these factors on anemia prevalence. Medical treatment is not the only driver of health of an individual, instead societal factors play a vital role in keeping a person healthy [17, 18]. Anemia instigates from a broader sociocultural background, whereby societal determinants are of recognized importance for understanding the causal association of society and this ailment. Furthermore, these studies were conducted on different age ranges of adolescent girls, and they were not of standard age category (10 and 19 years).

The present study is aimed to estimate the prevalence of anemia among adolescent girls (aged 10 to 19 years) in the Muzaffarabad division of AJK and to determine societal factors associated with it. The outcomes of the study can be helpful for devising effective interventions for improving nutritional status of adolescent girls to prevent occurrence of anemia and its mental, physical, and reproductive health consequences.

## 2. Materials and Methods

### 2.1. Study Design

A cross-sectional study was conducted for assessing anemia prevalence and its societal factors among adolescent school girls. The study and its ethical clearance were approved by the board of advanced studies and research of International Islamic University. Informed consent of the respondents and their parents was also obtained before interview. The purpose of the study was orally explained to the respondents and their mothers. Confidentiality of the information and privacy of the respondents were also maintained.

### 2.2. Study Area

The study was conducted in the Muzaffarabad division, Azad Jammu and Kashmir (AJK), Pakistan, which comprises mountainous topography. AJK has three divisions and 10 districts [19, 20], and Muzaffarabad division was randomly selected for this study. The government and private-sector jobs, small businesses, livestock, tourism, horticulture, and collection of medicinal herbs are the main sources of livelihood. The region is badly affected by natural and manmade hazards. Because of limited job opportunities in the government and private sectors, a majority of people are working in Pakistan and abroad. Natural disasters and the firing across the line of control between Pakistani and Indian army has badly affected infrastructure, tourism, and hence the economic well-being of the people. Poverty and inadequate health facilities at far flung areas, insufficient diet, unawareness about anemia, and its health consequences make the female population vulnerable to iron deficiency anemia (IDA).

### 2.3. Sample Size and Sampling Procedure

The sample size was computed using the projected population of Muzaffarabad division for females aged 10 to 49 years in 2015 based on 1998 census. The population of female aging 10 to 15 years was *N* = 342150. The following formula [21] was used to compute the sample size:

(1) $$\begin{array}{c} n=\frac{\chi^{2}NP\left(1-P\right)}{d^{2}\left(N-1\right)+\chi^{2}P\left(1-P\right)}. \end{array}$$

The sample size of female aged 10 to 49 years was 1529 by considering the parameters *χ*^2^=3.841, *P*=0.50 (population proportion), *d*=0.025 (marginal error), and *N* = 342150. The final sample size of adolescent girls (10 to 19 years) was 626, which were 40.95% of the sample size of 10 to 49 years. The respondents were selected using multistage stratified proportionate random sampling technique. Among the three divisions of AJK, Muzaffarabad division was randomly selected. The two districts of Muzaffarabad division, Hattian Bala and Muzaffarabad, were then randomly selected. In the next stage, 4 and 8 union councils of Hattian Bala and Muzaffarabad districts were randomly selected, respectively. There were 8 and 16 union councils in the Hattian and Muzaffarabad districts, respectively, and we selected 50% of them from each district. The houses having at least one adolescent were randomly selected in each union council based on the proportional allocation. Adolescents who were either married or were suffering from known chronic illness were excluded from the study.

### 2.4. Data Collection

Data were collected using a pretested interview schedule, which comprised questions related to socioeconomic, demographic, cultural, and nutritional variables. To ensure data quality, prefield activities such as proper question ordering, time and relevancy of questions, training and field supervision, and pretesting were performed before data collection. Mechanical weighing scale with a height rod was used for measuring weight and height of the respondents. The weight was measured in kilograms (kg) and height in centimeters (cm), which was then converted into meters (m). Hemoglobin (Hb) level of the respondents was measured using a precalibrated instrument (HemoCue Hb 301 analyzer manufactured by Ängelholm, Sweden). Microcuvettes were used for taking capillary blood, which were then inserted into the HemoCue Hb analyzer for determining the Hb level. For adolescent girls aged 10–11 years, Hb ≤8.0 g/dL, 8.1 to 10.9 g/dL, 11 to 11.4 g/dL, and ≥11.5 g/dL was taken as severe, moderate, mild anemic, and normal, respectively, whereas for girls aged ≥12 years, Hb less ≤8.0 g/dL, 8.1 to 10.9 g/dL, 11 to 1.9 g/dL, and ≥12 g/dL was taken as severe, moderate, mild anemic, and normal, respectively [5]. Hb levels were adjusted for the altitude of residence of the respondents [5, 22].

### 2.5. Data Analysis

Data were checked, coded, cleaned, and entered in Statistical Package of Social Sciences (SPSS) version 14. The independent variables are the societal determinants of anemia, whereas the dependent variables include anemia determined based on the Hb level. Univariate analysis was performed to compute descriptive statistics for societal determinants of anemia and anemia prevalence. Chi-square test and odds ratio (OR) were used to explore the association of anemia with its societal determinants. Binary logistic regression was used for computing OR. The results were considered statistically significant for significance level ≤0.05. Multiple linear regression (MLR) was used to find the relationships of different socioeconomic, demographic, cultural, and nutritional variables (independent variables) with hemoglobin level (dependent variable) of the respondents. MLR was first used by Pearson in 1908 for assessing the association of more than one independent variable and a dependent variable [23]. MLR model is indicated using the following equation:

(2) $$\begin{array}{c} \hat{Y}=b_{o}+\overset{p}{\underset{i=1}{\sum }}b_{i}X_{i}, \end{array}$$

where $\hat{Y}$ is the predicted value of the dependent variable, *p* is the order of polynomial, *X*'s represent the independent variables, and *b*_*o*_ is the value of $\hat{Y}$, when all *X*'s are zero and *b*'s are the regression coefficients. Before using MLR, critical assumptions are satisfied for ensuring validity of the model. Normality assumption is checked by normal probability-probability (P-P) plot of regression standardized residuals, and homoscedasticity assumption is examined by constructing the scatterplot of standardized residual predicted values against the standardized residuals [24]. A random spread suggests that variance of residual is constant at each point of the model, which validates the assumption homoscedasticity [25, 26]. Collinearity assumption is tested using collinearity statistics tolerance and variance inflation factor (VIF). The assumption of collinearity is satisfied if tolerance >0.1 and VIF should be <10 [24]. The Durbin–Watson statistic is used for testing the assumption that residuals are uncorrelated, and Cook's distance is used to check the assumption that there are no influential cases biasing the model. After satisfying MLR assumptions, stepwise regression technique, which combines advantages of forward and backward selection [27], is used for data analysis. Stepwise regression uses an automatic procedure for the selection of predictive variables [27]. This technique initially starts from no variable and adds a predictor variable whose addition is a good fit for the model, and the procedure is repeated till further inclusion of variables do not improve the model significantly.

## 3. Results

### 3.1. Descriptive and Bivariate Analysis

Table 1 shows the distribution of adolescent girls according to anemia severity determined based on the hemoglobin level. A little more than half (52.1%) respondents were nonanemic, and 47.9% were suffering from anemia, which indicates that anemia is a severe health problem among adolescent girls from the public health perspective. The prevalence of mild, moderate, and severe anemia is 20.4%, 24.8%, and 2.7%, respectively. The mean ± standard deviation (SD) of the hemoglobin level for the nonanemic girls was 13.13 ± 0.90 grams/deciliter (g/dL), and for those suffering from mild anemia was 11.44 ± 0.31 g/dL, moderate anemia 10.03 ± 0.81 g/dL, and severe anemia 7.18 ± 5.09 g/dL. The mean hemoglobin level among all the adolescent girls was 11.86 ± 1.69 g/dL.

In Table 2, results of descriptive and bivariate analysis to reveal the individual characteristics and their association with the prevalence of anemia among adolescent girls are presented. The results aver that respondent's education, menstruation duration, heavy blood loss during menstruation, communicable diseases, healthcare utilization, meals regularity, and exercise habits were significantly associated with anemia among adolescent girls. Regarding educational attainment, majority of girls (33.1%) were students of 6^th^ to 8^th^ standard, followed by 29.7% of 9^th^ to 10^th^ standard, 28.4% were above 10^th^ standard, 8.1% of 1 to 5^th^ standard, and only 0.6% were illiterate. This reveals that almost 100% girls were enrolled in the schools for pursuing their education. The prevalence of anemia was lowest among the girls who had 10+ years of schooling. Majority of girls (36.6) had longer menstrual duration (5 + days): 36.4 had 4 to 5 days and 10.7% had 2 to 3 days, whereas 16.9 had no menstrual period. The odds of occurrence of anemia were 1.68 and 2.49 times among girls who had menstrual periods for 4 to 5 and 5 + days, respectively, compared with those who had no menstruation. Chi-square value 20.15 at a significance level <0.0001 reveals significantly high association between menstrual duration and anemia among girls. Heavy blood loss during periods was significantly associated with higher odds of anemia. The prevalence of communicable diseases among girls was 22% and was significantly associated with anemia (chi-square = 27.7 at significance level <0.0001). The visits for utilizing health care revealed significant association with the occurrence of anemia among girls. The odds of anemia are 3.35 times more among girls who did not utilize healthcare services compared with those who availed health care often. The prevalence of anemia was high among girls who were using food supplements. The utilization of food supplements was small among respondents, and those girls were using them who were already anemic. The current age; BMI; knowledge about balanced diet; utilization of iron supplements; and knowledge about anemia, its causes, and preventive measures did not reveal any significant association with anemia among girls.

Table 3 presents the results of descriptive and bivariate analyses to reveal the parental and community factors of anemia and their association with occurrence of anemia among adolescent girls. Regarding the parental education, both father's and mother's education were significantly associated with anemia among girls. The odds of occurrence of anemia were 61% less for the girls whose parents had more than 12 years of education. The economic well-being of respondents determined based on the father's, mother's, and family monthly income revealed significant association with anemia among girls. The odds of anemia were 2%, 39%, 54%, and 86% less for the adolescent girls whose father's monthly income was 20001 to 50000, 50001 to 100000, and 100000+ Pakistani rupees, respectively, compared with girls whose fathers were not earning. The odds of occurrence anemia were high for girls whose mothers had low-paid jobs; however, the odds of occurrence of anemia were less for girls, whose mother had high-paid jobs compared with housewives. The family monthly income was also associated with lowers odds of anemia occurrence among girls. The parental professions were significantly associated with anemia among girls. The findings aver that anemia was noticeably low among adolescent girls of government employees. Household structure was marginally associated with anemia among girls; however, provision of proper latrine facility at home did not reveal significant association with anemia among girls, but there was significant association of the provision of the sewerage system with anemia. The open sewerage system was significantly associated with anemia (chi-square 7.09 and significance level 0.008) among girls.

### 3.2. Multiple Linear Regression Analysis

**Figure 1:**
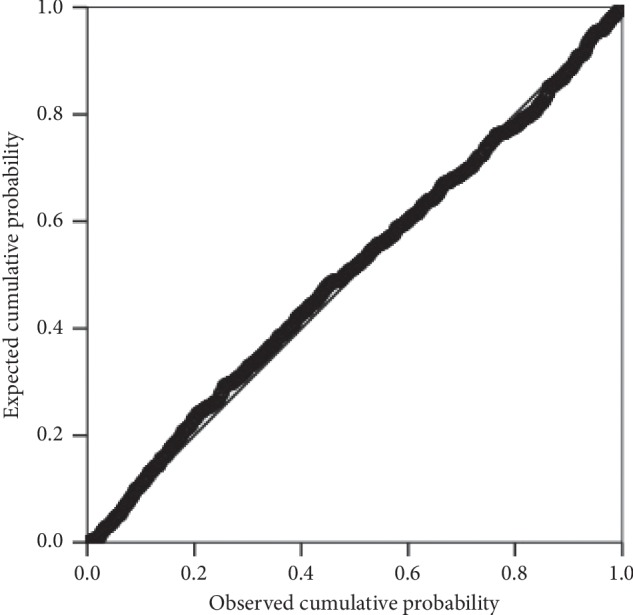
P-P plot of regression standardized residuals.

**Figure 2:**
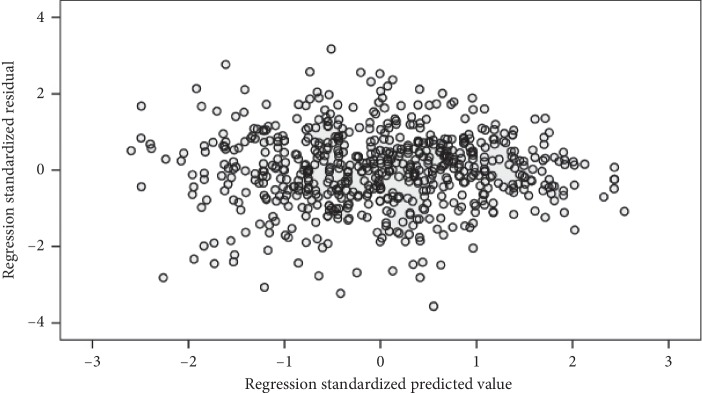
Scatterplot of regression standardized residuals.

Multiple linear regression (MLR) analysis is used to model the relationship of societal determinates (independent variables) and hemoglobin level (dependent variable) of the adolescent girls. Before performing MLR, we tested several assumptions to ensure reliability and validity of analyzed results. Only those independent variables were included in MLR analysis, which were significantly associated (*p* value ≤0.05) with dependent variables and obey the assumption of linearity. The assumption of normality is tested by looking at the P-P plots of residuals (Figure 1). It is evident from the figure that data points lie very close to the diagonal line, which indicates that residuals are normally distributed. The homoscedasticity assumption is examined by constructing the scatterplot of standardized residual predicted values against the standardized residuals [24]. The random array of dots (Figure 2) suggests that variation in residuals is similar at each point of the model, which validates the assumption of homoscedasticity.

The collinearity assumption is tested using two collinearity statistics tolerance and variance inflation factor (VIF). Tolerance <1 and VIF <2 indicate that data are not multicollinear. We used Durbin–Watson statistic for testing the assumption that residuals are uncorrelated (independent). The Durbin–Watson statistic varies between 0 and 4, and a value close to 2 renders the validity of the analysis. In this study, the value is 1.95 (very close to 2), which indicates that values of residuals are independent. Cook's distance is used to test the assumption that there are no influential cases biasing the MLR model. The Cook's distance >1 is an indicator of significant outlier, which can pose undue influence on the model that should be removed. In this study, Cook's distance is very less than 1 for each respondent; hence, there are no influential cases biasing the MLR model.

After testing assumptions, we used stepwise regression technique, which combines advantages of forward and backward selection [27] for MLR analysis. Stepwise regression uses automatic procedure for the selection of predictive variables, which are best fit for the MLR model and exclude the variables which do improve the model significantly [27]. In Table 4, results of the model summary and analysis of variance (ANOVA) are presented. The model summary (first part of the table) reveals the strength of the association between the MLR model and the dependent variable. The value of *R* is 0.36, which specifies a linear correlation between observed and predicted values of the hemoglobin level. The value of *R*^2^ (*R* square) is 0.131; this means that the model explains 13.1% variance in the hemoglobin level. In Table 5, the results to evaluate the contribution of each independent variable to predict the hemoglobin of the adolescent girls are shown.

(3) $$\begin{array}{c} \text{Hemoglobin}=11.123-0.666X_{1}-0.324X_{2}-0.252X_{3}-0.208X_{4}+0.400X_{5}+0.065X_{6}. \end{array}$$

The regression equation for predicting the hemoglobin level of adolescent girls from the independent variable is constructed using unstandardized coefficients (*B*) as detailed in the second column of Table 5.

In this model, communicable diseases and duration of menstruation have a significantly negative impact, whereas family monthly income, exercise habits, meals regularity, and respondent's education have a significantly positive impact on the hemoglobin level of the adolescent girls.

## 4. Discussion

Anemia in Pakistan is equally prevalent among both pregnant and nonpregnant women [7, 8]. Identifying subpopulations such as adolescent girls who are at high risk of anemia can assist to develop appropriate and effective interventions for preventing anemia during reproductive period. Anemia is the most prevalent micronutrient deficiency among adolescent girls in Pakistan [9–11]. In this study, we analyzed the societal determinants of anemia among adolescent girls to understand the causal association of society and this ailment. The findings of this study aver that anemia is severe public health problem among adolescent girls (47.9%) according to the WHO classification for public health significance. The prevalence of anemia among adolescent girls is relatively higher than that in the study [9] and is considerably low compared with that in the studies [10, 11] conducted in Pakistan.

The results of descriptive and bivariate analysis reveal that individual, parental, and community-based societal factors play a pivotal role in identifying girls at most risk of anemia. Individual determinants such education, duration of menstruation, heavy blood loss during menstruation, communicable diseases, healthcare utilization, meals regularity, and exercise habits showed a significant association with anemia among adolescent girls. The findings are in line with numerous studies conducted in Pakistan [25, 28–30]. Educated female can utilize adequate medical care and have appropriate knowledge about nutritional diet and personal hygiene [25].

Parental, household, and community factors are associated with anemia [25, 31–41]. The findings of our study elucidated that prevalence of anemia was smaller among girls whose parents had attained more than 12 years of education. The children of educated parents consume iron-rich food, utilize adequate healthcare facilities, and manage hygienic household environment, which can be associated with reduction in anemia among adolescent girls. The findings are consistent with the studies [31, 32], which reported that children of highly educated parents have lesser predisposition to develop anemia. Economic well-being of girls determined based on the father's, mother's, and family monthly revealed that all the independent variables are significantly associated with the prevalence of anemia among girls. The likelihood of anemia reduced substantially for the adolescent girls whose parental or family earnings were higher than those which had low income. The results are in good agreement with numerous studies conducted in different parts of the world and in Pakistan that economic well-being is a determinant factor of anemia among females [25, 33–38]. Our study highlighted that girls of government employees and businessmen are less vulnerable to develop anemia. The prevalence of anemia was low among girls whose mothers were government employees compared with that of anemia among girls whose mothers were with other professions. Similar results are reported by Kulkarni et al. [39] that occurrence of anemia is low among adolescent girls whose mothers are in service or doing business compared with housewives or laborers. Anemia prevalence was low among girls whose fathers are government employees, businessmen, or skilled laborers. This finding is concordant with the study conducted by Teji et al. [40] that nutritional status and occurrence of anemia among adolescent girls are directly associated with father's occupation. Poor household and environmental conditions are associated with anemia [41]. House structure and availability proper toilet were not associated with anemia among girls; however, anemia prevalence was comparatively more among girls residing in mud houses and do not have proper latrine at home. Higher prevalence of anemia was observed among girls residing in areas having an open sewerage system.

The societal determinants (predictors) that contributed to predict the hemoglobin level of adolescent girls in the MLR model are communicable diseases, menstrual duration, family monthly income, exercise habits, meals regularity, and respondent education. Strong association of anemia and communicable diseases was noted among adolescent girls. This finding highlights that prevention and treatment of communicable diseases is the matter of deep concern among adolescent girls in the study population. The results are congruent with the findings of Batool [25] that communicable diseases are contributing factor of the decreased hemoglobin concentration among Pakistani women. Menstrual disorders not only affect daily activities and quality of life but are also the major cause of anemia among adolescent girls [28–30]. Numerous studies highlighted that family monthly income [25, 33–38], exercise [42, 43], meals regularity [25], and respondent education [25] are the societal determinants of anemia in female.

## 5. Conclusion

The present study explored the societal determinants of anemia among adolescent girls in Azad Kashmir, Pakistan. The findings documented that anemia is a severe public health problem among adolescent girls according to the United Nation's public health significance. Our findings confirm that anemia is a multifactor health problem that instigates from a broader background. Anemia among adolescent girls can be the predisposing factor of this health issue among women of reproductive age in Pakistan. Appropriate interventions intruded during adolescence can provide the best prospect to reduce anemia among women in Pakistan and to meet global target of 50% reduction in anemia among them by 2025. Respondent and her parental education and better socioeconomic status are directly linked to utilization of diverse and nutritious foods, utilization of adequate health care, better household, and environmental conditions, which can have important implications for preventing anemia. The intervention strategies should focus on prevention and early treatment of communicable disease and menstrual disorder, provision of nutritional education, creating awareness about meals regularity, and exercise habits for alleviating hemoglobin level among adolescent girls.

## Figures and Tables

**Table 1:** Prevalence of anemia and mean hemoglobin level.

Anemia severity	Number	Frequency (%)	Hemoglobin level
			Mean ± SD
Normal	326	52.1	13.13 ± 0.90
Mild	128	20.4	11.44 ± 0.31
Moderate	155	24.8	10.03 ± 0.71
Severe	17	2.7	7.18 ± 0.59
Total	626	100	11.86 ± 1.69

**Table 2:** Descriptive and bivariate analyses to reveal the individual characteristics and their association with prevalence of anemia among adolescent girls.

Variable	Category	Nonanemic	Anemic	Total	Chi-square	*p* value	Odds ratio
		number (%)	number (%)	number (%)			
Current age (years)	10 to 13	90 (56.6)	69 (43.4)	159 (25.4)	2.14	0.34	Reference
	14 to 16	124 (49.2)	128 (50.8)	252 (40.3)			1.35
	17 to 19	112 (52.1)	103 (47.9)	215 (34.3)			1.20

Educational attainment (years)	Zero	1 (75.0)	3 (25.0)	4 (0.6)	9.42	0.05	2.04
	1 to 5	26 (51.0)	25 (49.0)	51 (8.1)			1.42
	6 to 8	110 (53.1)	97 (46.9)	207 (33.1)			1.30
	9 to 10	83 (44.6)	103 (54.4)	186 (29.7)			1.83
	10+	106 (59.6)	72 (40.4)	178 (28.4)			Reference

BMI (kg/m^2^)	Underweight	75 (53.2)	66 (46.8)	141 (22.5)	1.19	0.76	Reference
	Normal	226 (51.1)	216 (49.9)	442 (70.6)			1.09
	Overweight	19 (55.9)	15 (44.1)	34 (5.4)			0.90
	Obese	6 (66.7)	3 (33.3)	9 (1.4)			0.57

Menstrual period duration	No	66 (64.7)	36 (35.5)	102 (16.3)	20.15	<0.0001	Reference
	2 to 3 days	44 (65.7)	23 (34.3)	67 (10.7)			0.96
	4 to 5 days	119 (52.2)	109 (47.8)	228 (36.4)			1.68
	5+ days	97 (42.4)	132 (57.6)	229 (36.6)			2.49

Heavy blood loss during menstruation	Yes	56 (40.6)	82 (59.5)	138 (22.0)	9.38	0.002	1.48
	No	270 (55.3)	218 (44.7)	488 (78.0)			Reference

Communicable diseases	Yes	70 (36.5)	122 (63.5)	192 (30.7)	27.7	<0.0001	2.51
	No	256 (59.0)	178 (41.0)	434 (69.3)			Reference

Healthcare utilization	Not at all	14 (32.6)	29 (67.4)	43 (6.9)	10.79	0.005	3.35
	Sometimes	244 (51.6)	229 (48.4)	473 (75.6)			1.52
	Often	68 (61.8)	42 (38.2)	110 (17.6)			Reference

Knowledge about balanced diet	Poor	133 (52.8)	119 (47.2)	252 (40.3)	0.08	0.77	0.95
	Good	193 (51.6)	181 (48.4)	374 (49.7)			Reference

Take meals regularly	Yes	267 (55.7)	212 (44.3)	479 (76.5)	10.98	0.001	Reference
	No	59 (40.1)	88 (59.9)	147 (23.5)			1.88

Use of iron supplements	Yes	20 (57.1)	15 (42.9)	35 (5.6)	0.38	0.54	Reference
	No	306 (51.8)	258 (48.2)	591 (94.4)			1.12

Use of food supplements	Yes	16 (36.4)	28 (63.6)	44 (7.0)	4.68	0.03	Reference
	No	310 (53.3)	272 (46.7)	582 (93.0)			0.50

Knowledge about anemia	Poor	60 (49.2)	62 (50.8)	122 (19.5)	0.51	0.48	1.15
	Good	266 (52.8)	238 (74.2)	504 (80.5)			Reference

Knowledge about anemia causes	Poor	139 (54.3)	117 (45.7)	256 (40.6)	0.86	0.36	0.86
	Good	187 (50.5)	183 (49.5)	370 (59.1)			Reference

Knowledge about preventive measures	Poor	93 (55.4)	75 (44.6)	168 (26.8)	0.99	0.32	0.84
	Good	233 (50.9)	225 (49.1)	458 (73.2)			Reference

Exercise habits	Not at all	139 (44.8)	171 (55.2)	310 (49.5)	14.55	0.001	1.15
	Sometimes	30 (68.2)	14 (31.8)	44 (7.0)			0.64
	Often	157 (57.7)	115 (42.3)	272 (43.5)			Reference

**Table 3:** Descriptive and bivariate analyses to reveal the parental and societal determinants of anemia and their association with the prevalence of anemia among adolescent girls.

Variables	Category	Nonanemic	Anemic	Total	Chi-square	*p* value	OR
		Number (%)	Number (%)	Number (%)			
Father's education	Zero	38 (48.7)	40 (51.3)	78 (12.5)	15.10	0.004	Reference
	1 to 5	37 (47.4)	41 (52.6)	78 (12.5)			1.05
	6 to 8	45 (46.4)	52 (51.3)	97 (15.5)			1.10
	9 to 12	143 (50.4)	141 (49.6)	284 (45.4)			0.94
	12+	63 (70.8)	26 (29.2)	89 (14.2)			0.39

Mother's education	Zero	89 (45.9)	105 (54.1)	194 (31.0)	13.43	0.009	Reference
	1 to 5	43 (47.3)	48 (52.7)	91 (14.5)			0.95
	6 to 8	57 (60.0)	38 (40.0)	95 (15.2)			0.57
	9 to 12	93 (51.1)	89 (48.9)	182 (29.1)			0.81
	12+	44 (68.7)	20 (31.3)	64 (10.2)			0.39

Father's monthly income	No income	33 (43.4)	43 (56.6)	76 (12.1)	19.84	0.001	Reference
	Up to 20000	88 (44.0)	112 (56.0)	200 (31.9)			0.98
	20001 to 50000	138 (55.9)	109 (44.1)	247 (39.5)			0.61
	50001 to 100000	50 (60.2)	33 (39.8)	83 (13.3)			0.46
	100000+	17 (85.0)	3 (15.0)	20 (3.2)			0.14

Mother's monthly income	No income	254 (50.3)	251 (49.7)	505 (80.3)	15.94	0.003	Reference
	Up to 10000	22 (46.8)	25 (53.2)	47 (7.5)			1.15
	10001 to 20000	11 (45.8)	13 (54.2)	24 (3.8)			1.20
	20001 to 50000	29 (74.4)	10 (25.6)	39 (6.2)			0.35
	50000+	10 (90.9)	1 (9.1)	11 (1.8)			0.10

Family monthly income	Up to 20000	69 (39.9)	104 (60.1)	173 (27.6)	28.27	<0.0001	Reference
	20001 to 50000	125 (49.4)	128 (50.6)	253 (40.4)			0.68
	50001 to 100000	95 (63.3)	55 (36.7)	150 (24.0)			0.38
	100000+	37 (74.0)	13 (26.0)	50 (8.0)			0.23

Father's profession	Government service	132 (57.1)	99 (42.9)	231 (36.9)	18.52	0.001	Reference
	Private service	58 (40.6)	85 (59.4)	143 (22.8)			1.95
	Businessman	64 (57.7)	47 (4.3)	111 (17.7)			0.95
	Skilled laborer	32 (66.7)	16 (33.3)	48 (7.7)			0.47
	Others	40 (43.0)	53 (57.0)	93 (14.9)			1.77

Mother's profession	House wife	246 (50.2)	244 (49.8)	490 (78.3)	18.45	0.001	3.19
	Government service	45 (76.3)	14 (23.7)	59 (9.4)			Reference
	Private service	8 (44.4)	10 (55.6)	18 (2.9)			4.02
	Self employed	13 (37.1)	22 (62.9)	35 (5.6)			5.44
	Others	14 (58.3)	10 (41.7)	24 (3.8)			2.33

Family size	Up to 4	22 (47.8)	24 (52.2)	46 (7.3)	4.54	0.10	Reference
	5 to 8	219 (55.3)	177 (44.7)	396 (63.3)			0.74
	8+	85 (46.2)	99 (53.8)	374 (29.4)			1.07

Preferred baby gender of parents	Son	64 (47.4)	71 (52.6)	135 (21.6)	2.14	0.34	Reference
	Daughter	70 (56.5)	54 (43.5)	124 (19.8)			0.70
	Does not matter	192 (52.3)	175 (47.7)	367 (58.6)			0.82

Preference in food intake	Male	26 (43.3)	34 (56.7)	60 (9.6)	2.03	0.36	Reference
	Female	96 (53.0)	85 (47.0)	181 (28.9)			0.68
	Both	204 (53.0)	181 (47.0)	385 (61.5)			0.68

Household structure	RCC	198 (52.9)	176 (47.1)	374 (59.7)	7.51	0.06	Reference
	Mud	5 (26.3)	14 (73.7)	19 (3.0)			3.15
	Wooden	5 (83.3)	1 (16.7)	6 (1.0)			0.23
	Shelter	118 (52.0)	109 (48.0)	227 (36.3)			1.04

Latrine facility	Yes	304 (52.3)	277 (47.7)	581 (92.8)	0.20	0.66	Reference
	No	22 (48.9)	23 (51.1)	45 (7.2)			1.15

Sewerage system	Open	38 (39.6)	58 (60.4)	96 (15.3)	7.09	0.008	Reference
	Underground	288 (54.3)	242 (45.7)	530 (84.7)			0.84

**Table 4:** Model summary and ANOVA statistics.

Model summary					
*R*		*R* ^2^	Standard error of estimates		Durbin–Watson
0.36		0.13	1.36		1.95

ANOVA statistics					
Model	Sum of squares	df	Mean square	*F*-measure	Significance

Regression	234.57	6	38.05	15.54	<0.0005
Residual	1557.29	619	2.53		
Total	1791.86	625			

**Table 5:** Association of socioeconomic and environmental variables with anemia severity.

Model	Unstandardized coefficients		Significance
	*B*	Standard error	
Constant	11.23	0.44	<0.0005
Communicable diseases (*X*_1_)	−0.67	0.14	<0.0005
Duration of menstruation (*X*_2_)	−0.32	0.67	<0.0005
Family monthly income (*X*_3_)	0.25	0.07	<0.0005
Exercise habits (*X*_4_)	0.21	0.66	0.002
Meals regularity (*X*_5_)	0.40	0.15	0.008
Respondent education (*X*_6_)	0.07	0.03	0.028

Excluded variables are father's education, mother's education, father's profession, father's monthly income, mother's monthly income, heavy blood loss, and healthcare utilization.

## Data Availability

Data used in this study are available from the corresponding author upon request.
